# Red blood cell distribution width as a simple negative prognostic factor in patients with diffuse large B-cell lymphoma: a retrospective study

**DOI:** 10.3325/cmj.2015.56.334

**Published:** 2015-08

**Authors:** Vlatka Periša, Lada Zibar, Jasminka Sinčić-Petričević, Ana Knezović, Igor Periša, Jerko Barbić

**Affiliations:** 1 Department of Hematology, Clinic of Internal Medicine, University Hospital Centre Osijek, Osijek, Croatia; 2 University Josip Juraj Strossmayer in Osijek, School of Medicine, Osijek, Croatia; 3 Department of Dialysis, Clinic of Internal Medicine, University Hospital Centre Osijek, Osijek, Croatia; 4 Community Health Centre Đakovo, Đakovo, Croatia; 5 Community Health Centre Vinkovci, Vinkovci, Croatia

## Abstract

**Aim:**

To determine the prognostic value of baseline red blood cell distribution width (RDW) in diffuse large B cell lymphoma (DLBCL) patients.

**Methods:**

Data from 81 DLBCL patients diagnosed from 2006 to 2013 at the University Hospital Center Osijek, Osijek, Croatia, were reviewed. We evaluated disease outcome, overall survival (OS) and event-free survival (EFS), and demographic, clinical and laboratory factors affecting outcome. Univariate analysis and Cox regression analysis were used.

**Results:**

Median age of patients was 64 years, 29 were men (35.8%). Higher RDW levels (%) were found in patients with advanced Ann Arbor clinical stage (14.94 ± 1.82 vs 13.55 ± 1.54, *P* = 0.001) and in those with poor response to therapy (14.94 ± 1.82 vs 13.55 ± 1.54, *P* = 0.001). Patients with RDW>15% (cut-off was calculated by receiver operating characteristics) had significantly worse OS (median [range], 33 months [20-46] vs 74 months [65-82], *P* < 0.001) and EFS (27 months [15-40] vs 68 months [59-77], *P* < 0.001). Cox regression analysis showed that RDW>15% was an independent prognostic factor for OS (HR 3.654, 95% CI 1.128-11.836) and EFS (HR 2.611, 95% CI 1.012-6-739).

**Conclusion:**

High baseline RDW is an independent prognostic marker of poor outcome in patients with DLBCL. RDW could be an easily available and inexpensive marker for the risk stratification in patients with DLBCL.

Red cell distribution width (RDW) is analyzed routinely as part of the complete blood count (CBC). It is a measure of heterogeneity of the red blood cell (RBC) size and traditionally has played a role in the differential diagnosis of anemia (1). High RDW values are associated with increased mortality in general population and in patients with cardiovascular disease, sepsis, acute kidney injury, chronic obstructive pulmonary disease, hepatitis B, and those on chronic dialysis (2-9). There is also evidence of its prognostic value in various malignancies (10-14). A recent study found a strong relation between high RDW and poor survival in patients with lung cancer (15). RDW was also found to be a significant predictor of poor prognosis in patients with malignant mesothelioma (16). In patients with symptomatic multiple myeloma, elevated RDW values were associated with a higher stage disease according to International Staging System and poor prognosis (17). The mechanism that could explain the relation between RDW and survival or disease activity is not clear, but it is considered that high RDW is caused by chronic inflammation, poor nutritional status, oxidative stress, and age-related diseases that lead to changes in erythropoiesis (2,18-20).

Diffuse large B-cell lymphoma (DLBCL) is the most common group of lymphomas, amounting to 25% of all non-Hodgkin’s lymphomas (NHL) (21). It is a type of aggressive lymphoma that usually affects middle-aged and elderly patients. The distribution of NHL subtypes in Croatia corresponds to the European average (22). The most commonly used prognostic index in aggressive NHL is the international prognostic index (IPI) and its variants used in elderly patients (age-adjusted IPI) and in patients treated with rituximab (R-IPI) (23,24).

So far, there have been no reports on the prognostic value of RDW in patients with DLBCL. The aim of our study was to determine whether RDW measured at diagnosis was an independent prognostic factor of disease outcome, overall survival (OS), and event-free survival (EFS) in patients with DLBCL.

## Methods

This retrospective study included registry data on 81 patients with histologically verified nodal and extranodal DLBCL, diagnosed between November 2006 and December 2013 at the tertiary care University Hospital Centre Osijek. The study included patients who were in clinical stage II-IV by Ann Arbor (AA) staging, or IE or I bulky shape, who were initially planned for at least 4 cycles of chemotherapy and who had complete clinical data. Exclusion criteria included transformed indolent lymphoma and primary DLBCL of the central nervous system (CNS).

The following Demographic characteristics, clinical features, and laboratory parameters were collected from medical records: AA stage, IPI, B symptoms, comorbidity, serum lactate dehydrogenase (LDH), serum C-reactive protein (CRP), serum albumin, RBC, serum hemoglobin concentration (Hgb), mean corpuscular volume (MCV), white blood cells count (WBC), platelet count, absolute lymphocyte count (ALC), serum ferritin, RDW, Eastern Cooperative Oncology Group performance status (ECOG PS), and response to therapy.

Disease staging was done according to the AA classification. Performance status was quantified using ECOG. Most of the patients (90%) were treated with standard immunochemoterapy: rituximab, cyclophosphamide, doxorubicin, vincristine, and prednisone (R-CHOP) regimen, and others by R-CHOP-like regimens.

The outcomes were response to treatment, EFS, and OS. In patients in whom treatment was initiated and completed, the response to the therapy was determined by the International Working Group response criteria (25). EFS started from the first day of diagnosis to one of the events: disease progression, initiation of another anti-lymphomas treatment, relapse, death due to any cause, or until the latest control. OS refereed to period from date of the diagnosis until the date of death due to any cause or to the date of the latest control.

Initial values of RDW and of other laboratory parameters were defined as values obtained within 2 weeks before a front line-treatment. CBC including RDW calculation was determined from whole blood with K2 EDTA or K3 EDTA as an anticoagulant on Adiva 2100 analyzer (Siemens Healthcare Diagnostics, Tarrytown, NY, USA). RDW values were categorized based on the reference range in our laboratory, which is between 9%-15%, ie, the patients were divided in two groups using the cut-off value of 15%. The cut off value obtained by receiver operating characteristics (ROC) analysis was also 15%.

### Statistical analysis

SPSS (version 15.0, SPSS Inc. Chicago, IL, USA) and MedCalc Statistical Software, version 11.4.2.0 (Ostend, Belgium) were used. Variables were tested for normality using Kolmogorov-Smirnov test. Continuous variables with normal distribution were expressed as mean ± standard deviation (SD) and those with not normal distribution as median and interquartile range (IQR). Categorical variables were compared by χ^2^ test or Fisher exact test. Two continuous independent variables were analyzed by *t* test for normally distributed variables and by non-parametric Mann-Whitney U test for not normally distributed variables. More than two independent samples were analyzed using one-way analysis of variance (ANOVA) or Kruskal-Wallis test. Correlation was assessed using Pearson or Spearman test, as appropriate. Survival was analyzed with Kaplan-Meier curves and survival variables were compared with log-rank test. To estimate the predictive value of RDW we used Cox regression univariate and multivariate analysis. ROC analysis was used to determine the cut-off value of RDW for mortality. *P* value <0.05 was considered statistically significant.

## Results

**Figure 1:**
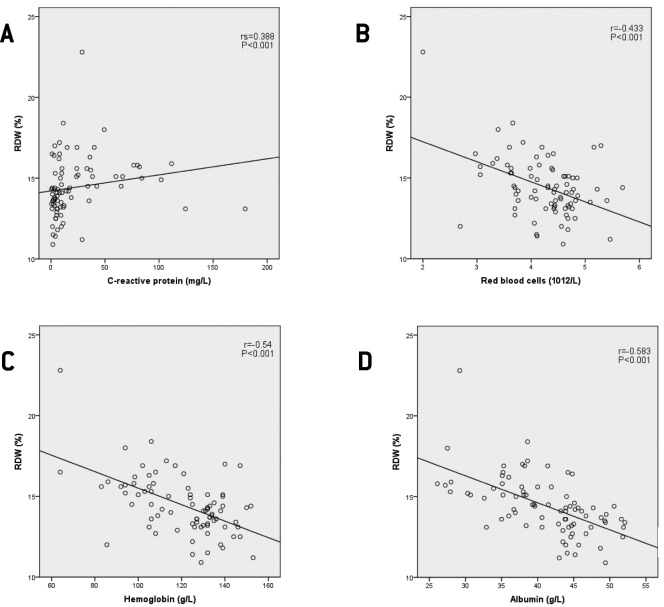
Correlation between red blood distribution width (RDW) and (**A**) C-reactive protein; (**B**) red blood cells count; (**C**) hemoglobin concentration; (**D**) albumin concentration in patients with diffuse large B-cell lymphoma.

**Figure 2:**
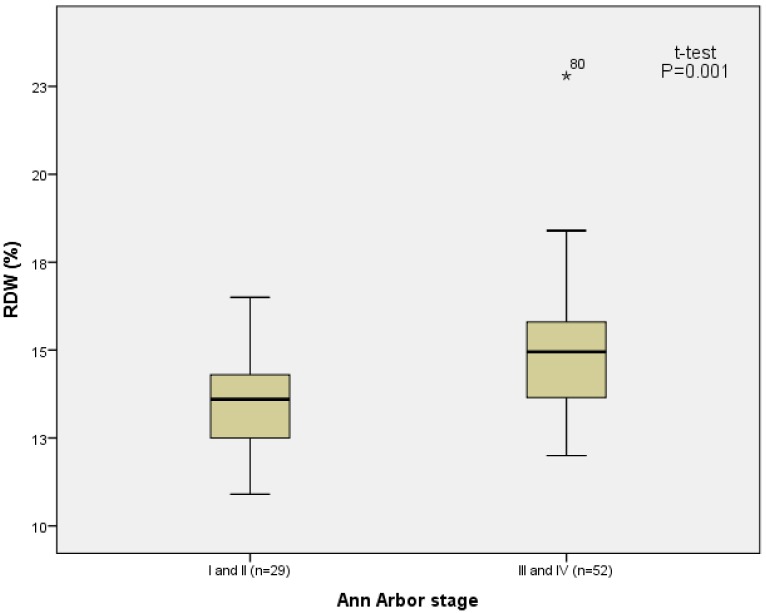
Baseline red blood cell distribution width (RDW). Patients with diffuse large B-cell lymphoma (N = 81) were divided according to Ann Arbor clinical staging.

The total number of eligible patients with DLBCL was 87. Six patients were excluded (2 who were in AA stage I, 1 due to insufficient clinical data, 1 due to transformed indolent lymphoma, and 2 for primary CNS disease). Fifty two patients were women and median age of all patients was 64 years (IQR 52.5-72.5 years). Median RDW was 14.3% (IQR 13.25%-15.55%). There was a significant positive correlation between RDW and CRP, IPI, ECOG PS, and clinical stage (r_S_ = 0.388, *P* < 0.001; r_S_ = 0.551, *P* < 0.001; r_S_ = 0.284, *P* = 0.01; r_S_ = 0.384, *P* = 0.001, respectively) and negative significant correlation between RBC, Hgb, and serum albumin concentration (r = -0.433, *P* < 0.001; r = -0.54, *P* < 0.001; r = -0.583, *P* < 0.001, respectively) (Figure 1). We found no correlation between RDW and WBC, ALC, platelet count, serum creatinine, and serum ferritin concentrations. Patients with higher AA stage (III and IV) had higher RDW values compared to patients with lower AA stages (stage I and II) (14.94 ± 1.82% vs 13.55 ± 1.54%, *P* = 0.001, *t* test, Figure 2).

**Figure 3:**
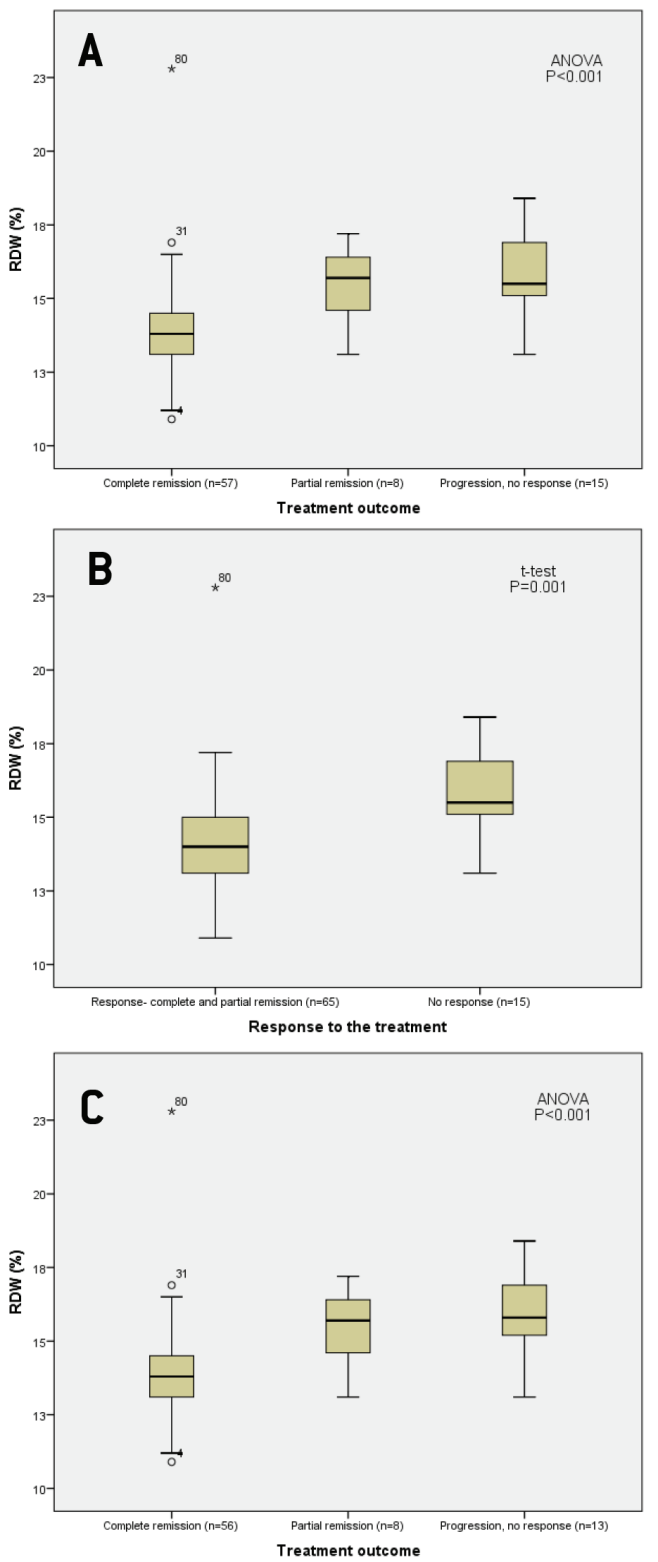
Baseline red blood cell distribution width (RDW) in patients with diffuse large B-cell lymphoma (DLBCL) (N = 80) (**A**) according to the treatment outcome and (**B**) according to response to the treatment; (**C**) in patients with DLBCL treated with immunochemotherapy according to the treatment outcome (N = 77).

RDW values differed significantly between groups according to treatment outcome (complete remission vs partial remission vs no response-progression, 13.93 ± 1.78 vs 15.46 ± 1.34 vs 15.77 ± 1.47, *P* < 0.001, ANOVA, Figure 3A). In patients treated with immunochemotherapy (N = 77) RDW values also differed significantly between groups according to treatment outcome (13.95 ± 1.79 vs 15.46 ± 1.34 vs 15.98 ± 1.44, *P* < 0.001, ANOVA, Figure 3C). Patients who responded to therapy had lower RDW values than those who did not respond to therapy (13.55 ± 1.54% vs 14.94 ± 1.82%, *P* = 0.001, *t* test, Figure 3B).

**Figure 4:**
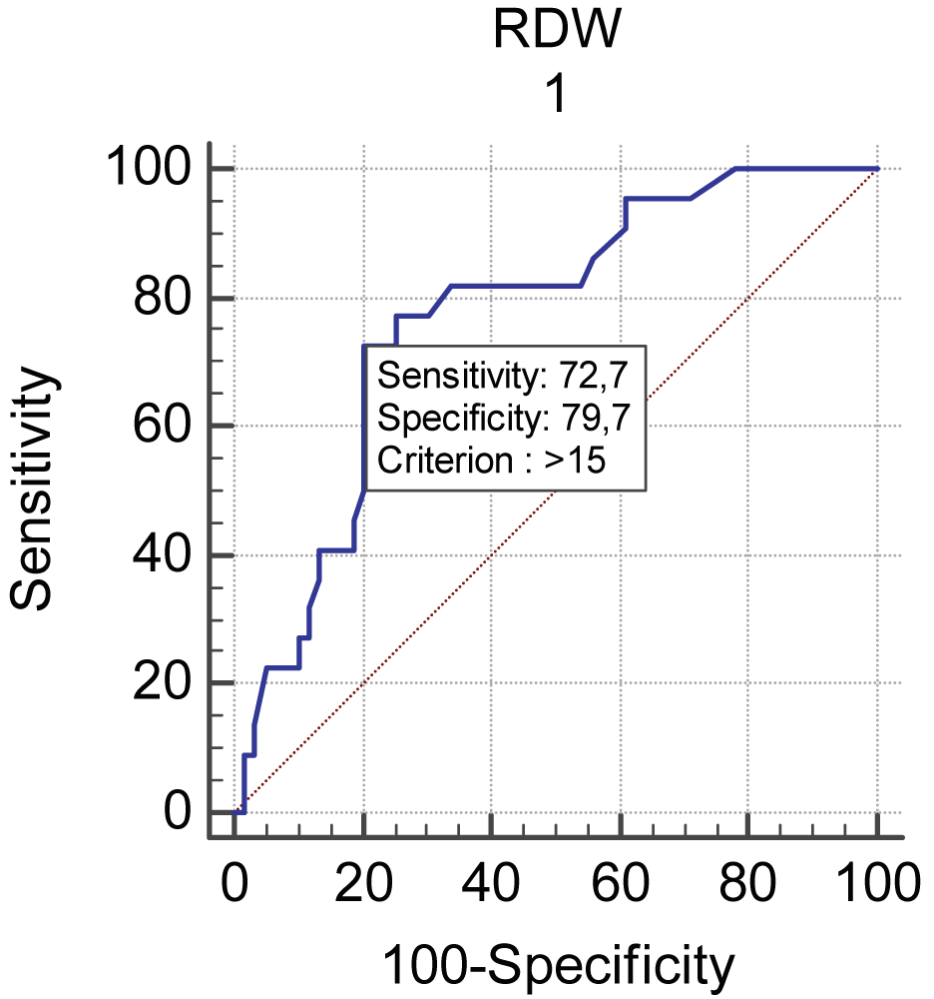
Receiver operating characteristic curve (ROC) of red cell distribution width (RDW) for differentiating overall survival in patients with diffuse large B-cell lymphoma (DLBCL) (N = 81).

According to both ROC analysis and the reference range for RDW in our laboratory, the patients were divided into two groups. Area under the curve (AUC) for RDW was 0.768 (95% CI 0.661-0.855, Z = 4.724), optimal cut-off value was 15%, with 72.7% sensitivity and 79.7% specificity, *P* < 0.001 (Figure 4). 53 patients had normal RDW (≤15%) and 28 patients had elevated RDW (>15%). The patients with elevated RDW had significantly lower ECOG PS (*P* < 0.001), higher AA stage (*P* = 0.004), higher IPI (*P* < 0.001), higher CRP (*P* = 0.001), lower RBC (*P* = 0.001), lower serum Hgb (*P* < 0.001), lower albumin concentration (*P* < 0.001), poorer response to treatment (*P* < 0.001), more frequent expressed B symptoms (*P* < 0.001), and had infiltration of bone marrow (*P* = 0.01), and more often were older than 60 years (*P* = 0.023). Front line treatment, comorbidity, and serum ferritin concentrations did not differ significantly between the groups (Table 1).

**Table 1:** Patient baseline characteristics (overall and divided according to 15% RDW cutoff)*

Variable	Overall	Divided by RDW (%)		*P*
		normal (≤15) (n = 53)	elevated (>15) (n = 28)	
RDW (%), mean±SD	14.44 ± 1.84	13.44 ± 1	16.35 ± 1.54	<0.001^†^
Age in years, median (IQR)	64 (52.5-72.5)	62 (50.5-75)	69 (62-72)	0.137^†^
Age group, n (%)				
≤60 years	31 (38.3)	25 (47.2)	6 (21.4)	0.023^‡^
>60 years	50 (61.7)	28 (52.8)	22 (78.6)	
Sex, n (%)				
Male	29 (35.8)	18 (34)	11 (39.3)	0.635^‡^
Female	52 (64.2)	35 (66)	17 (60.7)	
ECOG PS, n (%)				
<2	62 (76.5)	47 (88.7)	15 (53.6)	<0.001^‡^
≥2	19 (23.5)	6 (11.3)	13 (46.4)	
IPI, n (%)				
≤2	46 (56.8)	41 (77.4)	5 (17.9)	<0.001^‡^
>2	35 (43.2)	12 (22.6)	23 (82.1)	
LDH, n (%)				
normal	44 (54.3)	38 (71.7)	6 (24.4)	<0.001^‡^
>241 U/L	37 (45.7)	15 (28.3)	22 (78.6)	
B symptoms ^II^, n (%)				
no	37 (45.7)	32 (60.4)	5 (17.9)	<0.001^‡^
yes	44 (54.3)	21 (39.6)	23 (82.1)	
Bone marrow infiltration, n (%)^¶^				
no	51 (63.7)	39 (73.6)	12 (44.4)	0.01^‡^
yes	29 (36.3)	14 (26.4)	15 (55.6)	
Ann Arbor clinical stage, n (%)				
I and II	29 (35.8)	25 (47.2)	4 (14.3)	0.004^‡^
III and IV	52 (64.2)	28 (52.8)	24 (85.7)	
Front line treatment, n (%)**				
immunochemotherapy	77 (96.2)	51 (96.2)	26 (96.3)	>0.999^‡^
chemotherapy	3 (3.7)	2 (3.8)	1 (3.7)	
Comorbidity, n (%)				
diabetes mellitus	6 (7.4)	5 (9.4)	1 (3.6)	0.659^‡^
hypertension	22 (27.2)	16 (30.2)	6 (21.4)	0.399^‡^
cardiovascular disease	12 (14.8)	7 (13.2)	5 (17.9)	0.575^‡^
chronic lung disease	1 (1.2)	0 (0)	1 (3.6)	0.346^‡^
chronic liver disease	1 (1.2)	1 (1.9)	0 (0)	>0.999^‡^
malignancy	4 (4.9)	2 (3.8)	2 (7.1)	0.606^‡^
bleeding	2 (2.5)	0 (0)	2 (7.1)	0.117^‡^
RBC × 10^12^/L, mean±SD	4.25 ± 0.64	4.44 ± 0.52	3.89 ± 0.71	0.001^†^
Hemoglobin (g/L), mean±SD	120.83 ± 19.46	128.71 ± 13.78	105.91 ± 20.07	<0.001^†^
MCV (fL), mean±SD	85.78 ± 6.2	86.23 ± 6.27	84.93 ± 6.09	0.373^†^
WBC × 10^9^/L, mean±SD	7.18 ± 2.63	6.95 ± 2.38	7.61 ± 3.04	0.325^†^
ALC (cells ×10^9^/L), mean±SD	1.61 ± 0.93	1.7 ± 0.7	1.42 ± 0.73	0.099^†^
Platelet ×10^9^/L, mean±SD	262.49 ± 124.15	246.79 ± 118.56	292.21 ± 131.15	0.118^†^
CRP (mg/L), median (IQR)	9.4 (3.9-31)	6 (3.05-13.15)	24.55 (8.48-46.9)	0.001^§^
Albumin (g/L), mean±SD	41.03 ± 6.42	44.01 ± 4.77	35.38 ± 5.25	<0.001^†^
Iron (µmol/L), mean±SD	10.41 ± 6.73	11.73 ± 6.68	7.96 ± 6.2	0.016^†^
Ferritin (µg/L), median (IQR)	97.25 (49.65-268.1)	85.85 (46.3-168.15)	134.2 (64.1-513.53)	0.05^§^
Treatment outcome, n (%)**				
response	65 (81.2)	50 (94.3)	15 (55.6)	<0.001^‡^
no response	15 (18.8)	3(5.7)	12 (44.4)	

*RDW – red blood cell distribution width; SD – standard deviation; IQR – interquartile range; ECOG PS – Eastern Cooperative Oncology Group performance status; IPI – International prognostic index; LDH – lactate dehydrogenase; RBC – red blood cells; MCV – mean corpuscular volume; WBC – white blood cells; ALC – absolute lymphocyte count; CRP – C-reactive protein.

^†^*t* test.

^‡^χ^2^ test.

^§^Mann-Whitney U test.

^II^fever, night sweat, weight loss.

^¶^in one patient there was no information about bone marrow infiltration.

**in one patient the lethal outcome occurred before the planned treatment.

### Survival and prognostic factors

**Figure 5:**
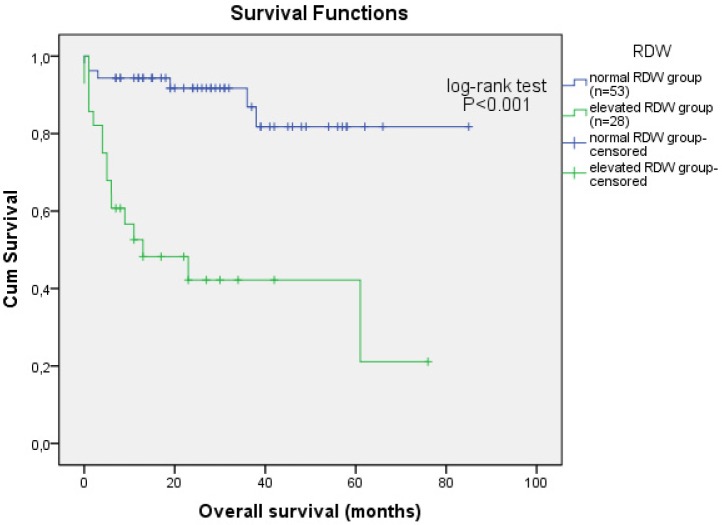
Survival curve for overall survival according to baseline red cell distribution width (RDW) (normal ≤15%, elevated >15%) in patients with diffuse large B-cell lymphoma (N = 81).

**Figure 6:**
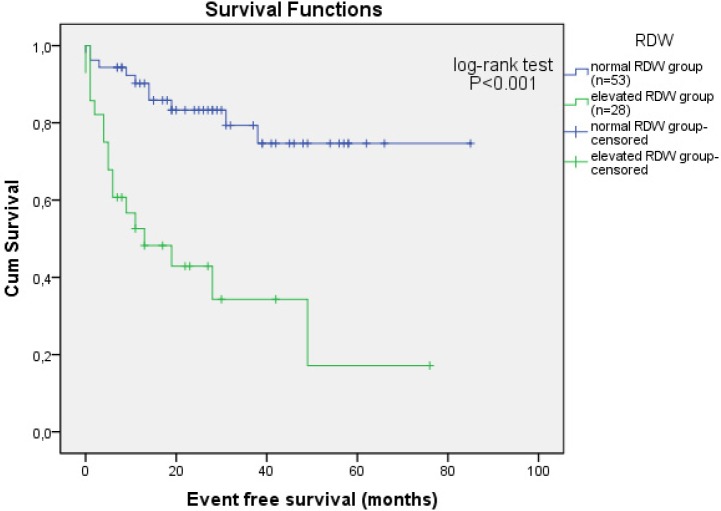
Survival curve for event free survival according to baseline red cell distribution width (RDW) (normal ≤15%, elevated >15%) in patients with diffuse large B-cell lymphoma (N = 81).

Median follow-up was 22 months (IQR 8.5-37.5 months), 22 (27.2%) patients died, and 27 (33.3%) experienced one of the events. 5-year OS was 67.9% for all patients, significantly lower in those with elevated RDW (36.6% vs 79.4%, *P* < 0.001, log-rank test) (Figure 5). Patients with elevated RDW had shorter expected OS than patients with normal RDW (33 months [20-46] vs 74 months [65-82], *P* < 0.001, log-rank test). 5-year EFS was 55.6% for all patients, significantly lower in those with elevated RDW (17.1% vs 74.7%, *P* < 0.001, log-rank test) (Figure 6). Patients with elevated RDW had shorter expected EFS than patients with normal RDW (27 months [15-40] vs 68 months [59-77], *P* < 0.001, log-rank test). Univariate Cox-regression analysis showed that prognostic factors for OS were elevated RDW levels (*P* < 0.001), age (*P* = 0.019), sex (male, *P* = 0.038), high ECOG PS (≥2, *P* < 0.001), high IPI (>2, *P* = 0.001), elevated LDH values (*P* = 0.002), and high clinical stage (stage III and IV, *P* = 0.011) (Table 2). Independent prognostic factors for EFS were elevated RDW level (*P* < 0.001), age (*P* = 0.006), high ECOG PS (≥2, *P* = 0.001), high IPI (>2, *P* < 0.001), elevated LDH values (*P* = 0.003), and high clinical stage (stage III and IV, *P* = 0.023) (Table 2). In the multivariate model RDW>15% was found to be an independent prognostic factor of OS (HR 3.654, 95% CI 1.128-11.386, *P* = 0.031) and EFS (HR 2.611, 95% CI 1.012-6.739) (Table 2). Lower ECOG PS was an independent prognostic factor for OS (HR 3.497, 95% CI 1.265-9.669, *P* = 0.016).

**Table 2:** Univariate and multivariate analysis for overall survival (OS) and event free survival (EFS) in patients with diffuse large B-cell lymphoma (N = 81)*

Univariate							Multivariate					
	OS		*P*	EFS		*P*	OS		*P*	EFS		*P*
	hazard ratio	95% confidence interval		hazard ratio	95% confidence interval		hazard ratio	95% confidence interval		hazard ratio	95% confidence interval	
RDW (%)	7.146	2.758-18.517	<0.001	5.026	2.273-11.113	<0.001	3.654	1.128-11.836	0.031	2.611	1.012-6.739	0.047
Age (year)	1.048	1.008-1.09	0.019	1.051	1.014-1.089	0.006	-	-	0.761	-	-	0.305
Sex (male)	2.737	1.051-5.652	0.038	-	-	0.076	-	-	0.14	-	-	-
ECOG PS (≥2)	6.202	2.518-15.278	<0.001	4.195	1.868-9.421	0.001	3.497	1.265-9.669	0.016	-	-	0.091
IPI (>2)	7.761	2.613-23.049	<0.001	5.387	2.261-12.835	<0.001	-	-	0.374	-	-	0.116
LDH (>241 U/L)	4.689	1.727-12.73	0.002	3.491	1.524-7.994	0.003	-	-	0.556	-	-	0.409
Clinical stage AA (III and IV)	6.623	1.546-28.378	0.011	3.097	1.170-8.203	0.023	-	-	0.488	-	-	0.974

*RDW – red blood cell distribution width; ECOG PS – Eastern Cooperative Oncology Group performance status; IPI – International prognostic index; LDH – lactate dehydrogenase; AA – Ann Arbor.

## Discussion

This study showed that increased RDW at diagnosis of DLBCL was associated with poor prognosis. To our knowledge, this is the first report on the prognostic value of RDW in patients with DLBCL. We showed that RDW was an independent prognostic factor for OS and EFS.

Elevated RDW was associated with poor PS, advanced clinical stage by AA, higher CRP, and lower albumin. The results support the idea that high levels of RDW reflect chronic inflammation and poor nutritional status in patients with DLBCL. Accordingly, there was a positive correlation between RDW and CRP and a negative correlation between RDW and albumin. To our knowledge, no study has investigated the association between malnutrition and RDW in patients with DLBCL. Therefore, the results of the present study are important, indicating significant association not only between RDW and CRP but also between RDW and hypoalbuminemia, which is indicative of malnutrition and mortality. It might be that inflammation and malnutrition impair erythropoiesis and thus contribute to the increase in RDW. Future studies are needed to elucidate if this, at least in part, explains the nature of the association between mortality and elevated RDW in patients with DLBCL. Earlier research found association between RDW and a variety of inflammatory markers, such as high sensitive CRP, erythrocyte sedimentation rate, IL-6, soluble transferrin receptor, soluble TNF receptor I, and soluble TNF receptor II (26). In our study, RDW value did not correlate with ferritin concentration. Patients with values of RDW>15% did not have significantly higher value of ferritin. This is an unexpected finding, because ferritin is one of the parameters of inflammation. However, in some patients with impaired liver function ferritin may not be a positive marker of inflammation. Except for albumin serum concentration, we did not analyze liver function. Similarly, in chronic hemodialysis patients significant association of CRP and albumin was found with RDW, but also lacking in correlation between RDW and ferritin (27). We found a positive association between clinical stage according to AA and RDW. This result might also reflect an association between RDW and increased inflammation or malnutrition caused by cancer progression.

Malignant tumors lead to chronic inflammation and malnutrition (28). This systemic inflammatory response, which reflects both disease activity and the host's innate response to tumor, may explain most symptoms and signs reported by cancer patients, including weight loss, anorexia, fatigue, and cancer related anemia (29). In different solid tumors, as well as lymphomas, inflammatory markers, including leukocytes, neutrophils, lymphocytes, CRP, and neutrophil/lymphocyte ratio have been associated with higher mortality rates (30-37). The mechanism that could explain the associations of RDW with survival or disease activity has not been elucidated, but possible explanation is that high levels of RDW reflect an underlying inflammatory state that impairs erythrocyte maturation and leads to inadequate production of the hormone erythropoietin, undernutrition (ie, deficiencies of nutrients, such as iron, vitamin B12, and folate), or oxidative damage as well as age associated diseases via changes in erythropoiesis (38). Our results are in accordance with findings on important roles of inflammation and malnutrition in tumor progression. Patients with elevated RDW had a poor response to treatment. Chronic inflammation is also reported to lead to an unfavorable response to chemotherapy (39,40). Poor survival in patients with elevated RDW might be due to chronic inflammation itself, or lack of response to chemotherapy. More research is needed to explain the relationships of RDW with inflammation and response to cancer treatment.

Recently, a few studies investigated the prognostic value of RDW in patients with malignant disease. RDW was significantly higher in patients with breast cancer than in patients with fibroadenomas (10). RDW was useful in the differentiation of benign and malignant causes of biliary obstruction when using an optimized cut-off value (11). RDW was a significant prognostic factor in patients with malignant mesothelioma, prostate, lung cancer, and multiple myeloma (15-17,41). Our results are concordant with these findings. It is possible that RDW is a general prognostic factor, common in various diseases.

A limitation of the study is its retrospective design and the fact that it was conducted in a single center. The obtained cut-off value should be externally validated within independent cohorts of patients in a preferably prospective study. Our patients underwent heterogeneous treatment regimens, but when we excluded the patients who were treated only by chemotherapy we confirmed that RDW had prognostic significance for treatment outcome in patients with DLBCL. Despite the limitations, this is the first study showing the prognostic value of RDW in patients with DLBCL with a long follow-up period. In conclusion, RDW could be a new, easily accessible, and inexpensive biomarker for risk assessment in patients with DLBCL.

## References

[1] Weiss G, Goodnough LT (2005). Anemia of chronic disease.. N Engl J Med.

[2] Patel KV, Semba RD, Ferrucci L, Newman AB, Fried LP, Wallace RB (2010). Red cell distribution width and mortality in older adults: a meta-analysis.. J Gerontol A Biol Sci Med Sci.

[3] Forhecz Z, Gombos T, Borgulya G, Pozsonyi Z, Prohaszka Z, Janoskuti L (2009). Red cell distribution width in heart failure: prediction of clinical events and relationship with markers of ineffective erythropoiesis, inflammation, renal function, and nutritional state.. Am Heart J.

[4] Felker GM, Allen LA, Pocock SJ, Shaw LK, McMurray JJ, Pfeffer MA (2007). Red cell distribution width as a novel prognostic marker in heart failure: data from the CHARM Program and the Duke Databank.. J Am Coll Cardiol.

[5] Jo YH, Kim K, Lee JH, Kang C, Kim T, Park HM (2013). Red cell distribution width is a prognostic factor in severe sepsis and septic shock.. Am J Emerg Med.

[6] Oh HJ, Park JT, Kim JK, Yoo DE, Kim SJ, Han SH (2012). Red blood cell distribution width is an independent predictor of mortality in acute kidney injury patients treated with continuous renal replacement therapy. Nephrology, dialysis, transplantation.

[7] Seyhan EC, Ozgul MA, Tutar N, Omur I, Uysal A, Altin S (2013). Red blood cell distribution and survival in patients with chronic obstructive pulmonary disease.. Journal of Chronic Obstructive Pulmonary Disease..

[8] Lou Y, Wang M, Mao W (2012). Clinical usefulness of measuring red blood cell distribution width in patients with hepatitis B.. PLoS ONE.

[9] Sicaja M, Pehar M, Derek L, Starcevic B, Vuletic V, Romic Z (2013). Red blood cell distribution width as a prognostic marker of mortality in patients on chronic dialysis: a single center, prospective longitudinal study.. Croat Med J.

[10] Seretis C, Seretis F, Lagoudianakis E, Gemenetzis G, Salemis NS (2013). Is red cell distribution width a novel biomarker of breast cancer activity? Data from a pilot study.. Journal of Clinical Medicine Research..

[11] Beyazit Y, Kekilli M, Ibis M, Kurt M, Sayilir A, Onal IK (2012). Can red cell distribution width help to discriminate benign from malignant biliary obstruction? A retrospective single center analysis.. Hepatogastroenterology.

[12] Baicus C, Caraiola S, Rimbas M, Patrascu R, Baicus A (2011). Utility of routine hematological and inflammation parameters for the diagnosis of cancer in involuntary weight loss. Journal of Investigative Medicine.

[13] Ozkalemkas F, Ali R, Ozkocaman V, Ozcelik T, Ozan U, Ozturk H (2005). The bone marrow aspirate and biopsy in the diagnosis of unsuspected nonhematologic malignancy: a clinical study of 19 cases.. BMC Cancer.

[14] Spell DW, Jones DV, Harper WF, David Bessman J (2004). The value of a complete blood count in predicting cancer of the colon.. Cancer Detect Prev.

[15] Koma Y, Onishi A, Matsuoka H, Oda N, Yokota N, Matsumoto Y (2013). Increased red blood cell distribution width associates with cancer stage and prognosis in patients with lung cancer.. PLoS ONE.

[16] Abakay O, Tanrikulu AC, Palanci Y, Abakay A (2014). The value of inflammatory parameters in the prognosis of malignant mesothelioma.. J Int Med Res.

[17] Lee H, Kong SY, Sohn JY, Shim H, Youn HS, Lee S (2014). Elevated red blood cell distribution width as a simple prognostic factor in patients with symptomatic multiple myeloma. BioMed Research International.

[18] Douglas SW, Adamson JW (1975). The anemia of chronic disorders: studies of marrow regulation and iron metabolism.. Blood.

[19] Ferrucci L, Guralnik JM, Woodman RC, Bandinelli S, Lauretani F, Corsi AM (2005). Proinflammatory state and circulating erythropoietin in persons with and without anemia.. Am J Med.

[20] Hunziker S, Celi LA, Lee J, Howell MD (2012). Red cell distribution width improves the simplified acute physiology score for risk prediction in unselected critically ill patients.. Crit Care.

[21] Swerdlow SH, Campo E, Harris NL, Jaffe ES, Pileri SA, Stein H, et al. WHO classification of tumours of haematopoietic and lymphoid tissues. 4th edt. Lyon: IARC Press; 2008.

[22] Dominis M. Malignant lymphoma – epidemiological situation in Croatia. [CD-ROM] 4th Roche's oncology weekend. Zagreb: Roche; 2007.

[23] (1993). A predictive model for aggressive non-Hodgkin's lymphoma. The International Non-Hodgkin's Lymphoma Prognostic Factors Project.. N Engl J Med.

[24] Sehn LH, Berry B, Chhanabhai M, Fitzgerald C, Gill K, Hoskins P (2007). The revised International Prognostic Index (R-IPI) is a better predictor of outcome than the standard IPI for patients with diffuse large B-cell lymphoma treated with R-CHOP.. Blood.

[25] Cheson B, Pfistner B, Juweid M, Gascoyne R, Specht L, Horning S, International Harmonization Project on Lymphoma (2007). Revised response criteria for malignant lymphoma.. J Clin Oncol.

[26] Lippi G, Targher G, Montagnana M, Salvagno GL, Zoppini G, Guidi GC (2009). Relation between red blood cell distribution width and inflammatory biomarkers in a large cohort of unselected outpatients.. Arch Pathol Lab Med.

[27] Tekce H, Tekce B, Aktos G, Tarisev M, Sit M (2014). The evaluation of red cell distribution width in chronic hemodialysis patients.. Int J Nephrol.

[28] Mantovani A, Allavena P, Sica A, Balkwill F (2008). Cancer-related inflammation.. Nature.

[29] Moore MM, Chua W, Charles KA, Clarke SJ (2010). Inflammation and cancer: causes and consequences.. Clin Pharmacol Ther.

[30] Cao Y, Shi YX, Chen JO, Tan YT, Cai YC, Luo HY (2012). Serum C-reactive protein as an important prognostic variable in patients with diffuse large B cell lymphoma. Tumour Biol.

[31] Mohri Y, Tanaka K, Ohi M, Yokoe T, Miki C, Kusunoki M (2010). Prognostic significance of host- and tumor-related factors in patients with gastric cancer.. World J Surg.

[32] Cox MC, Nofroni I, Laverde G, Ferrari A, Amodeo R, Tatarelli C (2008). Absolute lymphocyte count is a prognostic factor in diffuse large B-cell lymphoma.. Br J Haematol.

[33] Cox MC, Nofroni I, Ruco L, Amodeo R, Ferrari A, La Verde G (2008). Low absolute lymphocyte count is a poor prognostic factor in diffuse-large-B-cell-lymphoma.. Leuk Lymphoma.

[34] Oki Y, Yamamoto K, Kato H, Kuwatsuka Y, Taji H, Kagami Y (2008). Low absolute lymphocyte count is a poor prognostic marker in patients with diffuse large B-cell lymphoma and suggests patients' survival benefit from rituximab.. Eur J Haematol.

[35] Kim DH, Baek JH, Chae YS, Kim YK, Kim HJ, Park YH (2007). Absolute lymphocyte counts predicts response to chemotherapy and survival in diffuse large B-cell lymphoma.. Leukemia.

[36] Porrata LF, Ristow K, Habermann TM, Witzig TE, Inwards DJ, Markovic SN (2009). Absolute lymphocyte count at the time of first relapse predicts survival in patients with diffuse large B-cell lymphoma.. Am J Hematol.

[37] Porrata LF, Ristow K, Habermann T, Inwards DJ, Micallef IN, Markovic SN (2010). Predicting survival for diffuse large B-cell lymphoma patients using baseline neutrophil/lymphocyte ratio.. Am J Hematol.

[38] Evans TC, Jehle D (1991). The red blood cell distribution width.. J Emerg Med.

[39] Ho SY, Guo HK, Chen HH, Peng CJ (2003). Nutruitional predictors of survival in terminally ill cancer patients.. J Formos Med Assoc.

[40] Gibbs J, Cull W, Henderson W, Daley J, Hur K, Khuri SF (1999). Preoperative serum albumin as a predictor of operative mortality and morbidity: results from the National VA Surgical Risk Study.. Arch Surg.

[41] Albayrak S, Zengin K, Tanik S, Bakirtas H, Imamoglu A, Gurdal M (2014). Red cell distribution width as a predictor of prostate cancer progression.. Asian Pac J Cancer Prev.

